# Association between maternal depression and lower urinary tract symptoms in their primary school-age daughters: A birth cohort study

**DOI:** 10.1097/WON.0000000000001039

**Published:** 2024

**Authors:** Shayna D. Cunningham, Sarah Lindberg, Carol Joinson, David Shoham, Haitao Chu, Diane Newman, Neill Epperson, Linda Brubaker, Lisa Low, Deepa R. Camenga, D. Yvette LaCoursiere, Melanie Meister, Kimberly Kenton, Siobhan Sutcliffe, Alayne D. Markland, Sheila Gahagan, Tamera Coyne-Beasley, Amanda Berry, Participating PLUS Research Consortium Research Centers and Investigators at the time of this writing are as follows, Linda Brubaker, Elizabeth R. Mueller, Marian Acevedo-Alvarez, Colleen M. Fitzgerald, Cecilia T. Hardacker, Jeni Hebert-Beirne, Missy Lavender, David A. Shoham, Kimberly Sue Kenton, James W. Griffith, Melissa Simon, Julia Geynisman-Tan, Alayne D. Markland, Tamera Coyne-Beasley, Kathryn L. Burgio, Cora E. Lewis, Gerald McGwin, Camille P. Vaughan, Beverly Rosa Williams, Emily S. Lukacz, Sheila Gahagan, D. Yvette LaCoursiere, Jesse Nodora, Janis M. Miller, Lisa Kane Low, Bernard L. Harlow, Kyle D. Rudser, Sonya S. Brady, Haitao Chu, Cynthia S. Fok, Peter Scal, Todd Rockwood, Sarah Lindberg, Diane K. Newman, Ariana L. Smith, Amanda Berry, Andrea Bilger, Heather Klusaritz, Terri Lipman, Ann E. Stapleton, Jean F. Wyman, Siobhan Sutcliffe, Aimee S. James, Jerry L. Lowder, Melanie R. Meister, Leslie M. Rickey, Deepa R. Camenga, Shayna D. Cunningham, Linda Brubaker, Julia Barthold

**Affiliations:** Loyola University Chicago, Maywood, IL (U01DK106898); Loyola University Chicago, Maywood, IL (U01DK106898); Loyola University Chicago, Maywood, IL (U01DK106898); Loyola University Chicago, Maywood, IL (U01DK106898); Loyola University Chicago, Maywood, IL (U01DK106898); Loyola University Chicago, Maywood, IL (U01DK106898); Loyola University Chicago, Maywood, IL (U01DK106898); Loyola University Chicago, Maywood, IL (U01DK106898); Northwestern University, Chicago IL (U01DK126045); Northwestern University, Chicago IL (U01DK126045); Northwestern University, Chicago IL (U01DK126045); Northwestern University, Chicago IL (U01DK126045); University of Alabama at Birmingham, Birmingham, AL (U01DK106858); University of Alabama at Birmingham, Birmingham, AL (U01DK106858); University of Alabama at Birmingham, Birmingham, AL (U01DK106858); University of Alabama at Birmingham, Birmingham, AL (U01DK106858); University of Alabama at Birmingham, Birmingham, AL (U01DK106858); University of Alabama at Birmingham, Birmingham, AL (U01DK106858); University of Alabama at Birmingham, Birmingham, AL (U01DK106858); University of California San Diego, La Jolla, CA (U01DK106827); University of California San Diego, La Jolla, CA (U01DK106827); University of California San Diego, La Jolla, CA (U01DK106827); University of California San Diego, La Jolla, CA (U01DK106827); University of Michigan, Ann Arbor, MI (U01DK106893); University of Michigan, Ann Arbor, MI (U01DK106893); University of Minnesota, Scientific and Data Coordinating Center, Minneapolis MN (U24DK106786); University of Minnesota, Scientific and Data Coordinating Center, Minneapolis MN (U24DK106786); University of Minnesota, Scientific and Data Coordinating Center, Minneapolis MN (U24DK106786); University of Minnesota, Scientific and Data Coordinating Center, Minneapolis MN (U24DK106786); University of Minnesota, Scientific and Data Coordinating Center, Minneapolis MN (U24DK106786); University of Minnesota, Scientific and Data Coordinating Center, Minneapolis MN (U24DK106786); University of Minnesota, Scientific and Data Coordinating Center, Minneapolis MN (U24DK106786); University of Minnesota, Scientific and Data Coordinating Center, Minneapolis MN (U24DK106786); University of Pennsylvania, Philadelphia, PA (U01DK106892); University of Pennsylvania, Philadelphia, PA (U01DK106892); University of Pennsylvania, Philadelphia, PA (U01DK106892); University of Pennsylvania, Philadelphia, PA (U01DK106892); University of Pennsylvania, Philadelphia, PA (U01DK106892); University of Pennsylvania, Philadelphia, PA (U01DK106892); University of Pennsylvania, Philadelphia, PA (U01DK106892); University of Pennsylvania, Philadelphia, PA (U01DK106892); Washington University in St. Louis, Saint Louis, MO (U01DK106853); Washington University in St. Louis, Saint Louis, MO (U01DK106853); Washington University in St. Louis, Saint Louis, MO (U01DK106853); Washington University in St. Louis, Saint Louis, MO (U01DK106853); Yale University, New Haven, CT (U01DK106908); Yale University, New Haven, CT (U01DK106908); Yale University, New Haven, CT (U01DK106908); Steering Committee Chair; UCSD, San Diego. National Institute of Diabetes and Digestive and Kidney Diseases, Division of Kidney, Urologic, and Hematologic Diseases, Bethesda, MD; 1 Department of Public Health Sciences, University of Connecticut School of Medicine, Farmington, CT; 2 Division of Biostatistics, University of Minnesota School of Public Health, Minneapolis, MN; 3 Centre for Academic Child Health, Bristol Medical School, University of Bristol, Bristol, England; 4 Department of Biostatistics and Epidemiology, College of Public Health, East Tennessee State University, Johnson City, TN; 5 Division of Urology, Department of Surgery, Perelman School of Medicine, University of Pennsylvania, Philadelphia, PA; 6 Department of Psychiatry, University of Colorado, Aurora, CO; 7 Department of Obstetrics, Gynecology and Reproductive Sciences, University of California San Diego, San Diego, CA; 8 Department of Health Behavior and Biological Sciences, School of Nursing, University of Michigan, Ann Arbor, MI; 9 Department of Pediatrics, Yale School of Medicine, New Haven, CT; 10 Department of Obstetrics and Gynecology, University of Kansas, Kansas City, KS; 11 Department of Obstetrics and Gynecology, Northwestern University Feinberg School of Medicine, Chicago, IL; 12 Division of Public Health Sciences, Department of Surgery, and the Department of Obstetrics and Gynecology, Washington University School of Medicine, St. Louis, MO; 13 Department of Medicine and the Birmingham/Atlanta Geriatrics Research Education and Clinical Center, University of Alabama at Birmingham, Birmingham, AL; 14 Department of Pediatrics, University of California San Diego, La Jolla, CA; 15 Departments of Pediatrics and Internal Medicine, University of Alabama at Birmingham, Birmingham, AL; 16 Division of Urology, Children’s Hospital of Philadelphia, Philadelphia, PA

**Keywords:** Maternal depression, Daughters, Lower urinary tract symptoms, Avon Longitudinal Study of Parents and Children, urinary urgency, Overactive Bladder

## Abstract

**Purpose:**

Although maternal depression is associated with adverse outcomes in women and children, its relationship with lower urinary tract symptoms (LUTS) in offspring is less well characterized. We examined the association between prenatal and postpartum maternal depression and LUTS in primary school-age daughters.

**Design:**

Observational cohort study.

**Subjects and Setting:**

The sample comprised 7,148 mother-daughter dyads from the Avon Longitudinal Study of Parents and Children.

**Method:**

Mothers completed questionnaires about depressive symptoms at 18 and 32 weeks’ gestation and 21 months’ postpartum, and their children’s LUTS (urinary urgency, nocturia, and daytime and nighttime wetting) at 6, 7, and 9 years. Multivariable logistic regression models were used to estimate the association between maternal depression and LUTS in daughters.

**Results:**

Compared to daughters of mothers without depression, those born to mothers with both prenatal and postpartum depression had higher odds of LUTS, including urinary urgency (adjusted odds ratio [aOR] range 1.99-2.50) and nocturia (aOR range 1.67-1.97) at ages 6, 7, and 9 years. Additionally, daughters born to mothers with both prenatal and postpartum depression had higher odds of daytime wetting (aOR range 1.81-1.99) and nighttime wetting (aOR range 1.63-1.95) at 6 and 7 years. Less consistent associations were observed for depression limited to the prenatal or postpartum period only.

**Conclusions:**

Exposure to maternal depression in the prenatal and postpartum periods was associated with an increased likelihood of LUTS in daughters. This association may be an important opportunity for childhood LUTS prevention. Prevention strategies should reflect an understanding of potential biologic and environmental mechanisms through which maternal depression may influence childhood LUTS.

## Introduction

Lower urinary tract symptoms (LUTS) are common in early childhood; symptom prevalence diminishes with age, consistent with developmental maturation. ^1^ Neurodevelopmental risk factors, including developmental delay and child behavior/emotional difficulties, have been found to be prospectively associated with an increased risk of pediatric urinary incontinence. Population based studies have shown that both internalizing and externalizing disorders are more common in children with daytime incontinence and nocturnal enuresis.^2^ Urinary urgency is the most frequently reported LUTS in pediatric patients with attention deficit hyperactivity disorder (ADHD) symptoms.^3^ As neurologic maturation and function are necessary elements of bladder control, factors that modify neurodevelopment could also impact bladder health function.

Multiple studies have demonstrated an association between prenatal depression and increased risks of later behavioral and emotional problems in offspring.^4–6^ Postpartum, infancy and early childhood are sensitive periods when exposure to maternal depression may have a potentially adverse influence, acutely and over the life course. During the first year of life, the brain undergoes rapid growth and depends on stimulation and learning opportunities for optimal development.^7^ Maternal depression is associated with lower quality interactions between mother and child and less secure attachment, both of which may affect the child’s emotional and self-regulatory skills.^8^

Toilet training is a major developmental milestone requiring the right combination of child emotional, behavioral, and developmental skills and readiness within an environment that supports the training process.^9^ Toilet training and continence can be facilitated by maternal connectedness, sensitivity to the child’s cues, patience, positivity, and a relaxed demeanor,qualities that may be challenged in the setting of maternal depression.^8^ A recent study among a large British cohort found evidence that bith maternal prenatal and postpartum depression were associated with persistent (day and night) wetting in children ages 4 to 9 years old.^1^ Postpartum depression was also strongly associated with daytime wetting alone.^1^ Other studies have likewise reported a link between maternal affective disorders and bedwetting; however, these studies were limited to only lifetime psychopathology among mothers or exposure in the postpartum period.^10,11^ While day and nighttime wetting represent the extremes of lower urinary tract symptoms (LUTS), other childhood LUTS are important to consider. Urinary urgency is common in school age children, disproportionately affects girls, and is more often associated with daytime wetting in girls than boys.^12,13^ To date, studies have not examined how maternal depression may affect other LUTS such as urinary urgency or the potential cumulative effects of exposure during both the prenatal and postpartum periods.

The Prevention of Lower Urinary Tract Symptoms (PLUS) Research Consortium aims to develop strategies to prevent LUTS in girls and women across the life course.^14^ This study focuses on LUTS in girls. This study uses data from mother-daughter dyads in a large birth cohort to assess the association between maternal prenatal and postpartum depression with LUTS including urinary urgency, nocturia, daytime and nighttime wetting in primary school-age daughters.

## Methods

The Avon Longitudinal Study of Parents and Children (ALSPAC) included pregnant women residing in Avon, United Kingdom, with expected dates of delivery between April 1991 and December 1992. Detailed information about the cohort was collected since early pregnancy, including regular self-administered questionnaires from mothers and children. Information about ALSPAC is available at www.bristol.ac.uk/alspac/, including a searchable data dictionary (www.bristol.ac.uk/alspac/researchers/our-data/). Further details on the cohort profile, representativeness, and phases of recruitment are described in two cohort profile papers, ^15,16^ as well as an update.^17^ This analysis examined data from women with daughters alive at one year and information available on at least one LUTS outcome at ages 6 years 5 months (6 years), 7 years 9 months (7 years), or 9 years 7 months (9 years) (n=4,927) (see ‘Measures’ below for details of the LUTS variables).

Styduy precesdures were reviewed and approved by the ALSPAC ethics and law committee and local research ethics committees. Informed consent for the use of data collected via questionnaires was obtained from participants following the recommendations of the ALSPAC ethics and law committee at the time. As these analyses use pre-existing de-identified data, they do not constitute human subjects research or require additional approvals.

### Study Procedures and Instruments

Maternal depression was assessed at 18- and 32-weeks gestation and at 21 months postpartum, a peak period for initiation of toilet training in this cohort,^18^ using the Edinburgh Postpartum Depression Scale (EPDS).^19^ The EPDS was dichotomized at ≥13, a common cut-off used to indicate probable prenatal and postpartum depressive disorder.^20^ Mothers were categorized as having screened positive for prenatal depression if their EPDS scores were ≥13 at either 18- or 32-weeks’ gestation.

Parent-reported information on LUTS in the daughter was available at ages 6, 7, and 9 years. Items included: urgency (‘Does she have to dash to the toilet quickly when she realises she needs to go?’ yes, has to go straight away, can hold for a short time [less than 5 minutes], can hold for longer than 5 minutes);, nocturia (‘Frequency child gets up at night to go to toilet’ not at all, once, twice, 3 or more times), and urinary incontinence in the day and night (‘How often usually does your child wet herself during the day’ and ‘How often usually does your child wet the bed at night?’ never, less than once a week, about once a week, 2-5 times a week, nearly every day, and more than once a day). Urgency was categorized as “straight away” versus “can wait for any duration of time”; nocturia as “not at all” versus ‘ 1 or more times”; daytime wetting as “never”, “occasional but less than once a week” and “about once a week”, or “2 times a week or more”; and nighttime wetting as “never”, “weekly”, or “daily”.^21^

Potential confounders were selected based on literature review. A confounder is defined as a common cause of both exposure and outcome, but is not on the causal pathway from exposure to outcome. The analyses were adjusted for confounders assessed in the prenatal period. They were: 1.) maternal educational attainment (low: none, Certificate of Secondary School Education, or vocational; medium: high school qualifications obtained at age 16 years; high: advanced level qualifications obtained at age 18 years/degree or greater), 2.) home ownership status (owner versus renter), 3.) financial difficulties (yes versus no), 4.) family size (<3 children versus ≥3 children), and 5.) parental social class as determined during pregnancy and dichotomized into manual (partly or unskilled occupations) or non-manual (professional, managerial, or skilled professions) using the 1991 British Office of Population and Census Statistics classification.

### Data Analysis

Prior to analysis, multiple imputation was used to increase statistical power and reduce the risk of selection bias by imputing missing information for confounders and outcomes. Twenty datasets were generated using multiple imputation by chained equations and estimates of the association between maternal depression and LUTS in the daughter were obtained by averaging the results of 20 data sets using the Rubin rules. ^22,23^

Sensitivity analyses were also carried out by comparing imputed results with those from the complete-case analysis. As results in the average imputed analytic sample were similar to those in the pre-imputation sample (Appendix: Supplemental Digital Content), only results in the imputed sample are presented in the main text.

The association between maternal depression and LUTS in the daughter was estimated using multivariable logistic regression methods. As multivariable logistic regression models were fitted for four different outcomes at three different ages using data from the same participants, all p-values were adjusted for multiple testing by controlling the false discovery rate under dependency.^24^ Reported p-values were obtained for two-sided tests. All analyses were completed in SAS 9.4 mainly by procedures MI, GLIMMIX, MIANALYZE, and MULTITEST.^25^

## Results

The 15,454 participants in the ALSPAC study gave birth to 14,901 babies who were alive at one year of age, 7,148 (48.0%) of which were female. Of those, 4,927 (68.9%) had at least one outcome variable available at age 6, 7 or 9 and were included in the imputed analysis; 2,442 were in the complete case analysis (Appendix: Supplemental Digitl Content). Table 1 displays characteristics of mothers in the pre-imputation sample and the average imputed analytic sample. Home ownership was common (77.8%) and few (8.0%) reported financial difficulties. Maternal educational attainment was low in 26.8%, medium in 34.1%, and high in 39.2% of participants. Thirteen percent screened positive for depression in the prenatal period only, 3.6% in the postpartum period only, and 7.4% in both the prenatal and postpartum periods based on EPDS scores. Of the 645 mothers who screened positive for prenatal depression, 36.3% still screened positive for depression 21 months postpartum.

### Outcomes

The proportion of daughters with urinary urgency declined slightly with increasing age, from 10.4% at 6 years, 9.1% at 7 years and 8.3% at 9 years (Table 1). Nocturia remained stable over the time points measured. Occasional daytime wetting decreased from 10.4% at 6 years to 6.2% at 9 years, and the proportion of daughters without daytime wetting increased from 88.2% at 6 years to 93.2% at 9 years. The proportion of daughters with daytime wetting occurring twice per week or more remained stable over the first 2 time points (1.4% at 6 years, 1.6% at 7 years), but decreased by 9 years (0.7%). The frequency of nighttime wetting decreased for weekly and daily episodes from 6 to 9 years.

Table 2 summarizes associations between maternal depression and LUTS in their daughters. In unadjusted analyses, the presence of both prenatal and postpartum depression was associated with higher odds of LUTS in daughters at all ages examined: urinary urgency (odds ratio [OR] range 2.69-3.50), nocturia (OR range 2.19-2.60), daytime wetting (OR range 2.13-2.57), and nighttime wetting (OR range 2.33-2.53). Weaker and less consistent associations were observed for presence of prenatal depression only with daughters’ odds of urinary urgency (OR range 1.43-1.50 at 6 and 7 years), nocturia (OR range 1.35-1.51 at all ages), and daytime wetting (OR 1.50 at 9 years); and for postpartum depression only with daughters’ odds of urinary urgency (OR 1.89 at 7 years), daytime wetting (OR range 2.00-2.26 at 6 and 7 years), and nighttime wetting (OR 1.99 at 9 years).

Although controlling for potential confounders resulted in some attenuation of the odds ratios, there was still evidence for an association between both prenatal and postpartum depression and all LUTS examined in daughters. Social class and financial difficulty were the biggest drivers of attenuation in the adjusted model. The presence of both prenatal and postpartum depression was associated with urinary urgency (adjusted OR [aOR] range 1.99-2.50) and nocturia (aOR range 1.67-1.97) among daughters at each time point. Daytime and nighttime wetting were associated with the presence of both prenatal and postpartum depression at 6 and 7 years (daytime wetting: aOR 1.99, 95% confidence interval [CI] 1.4-2.8; aOR 1.81, 95% CI 1.2-2.8, respectively; nighttime wetting: aOR 1.95, 95% CI 1.4-2.7; aOR 1.63, 95% CI 1.1-2.3, respectively). In general, associations tended to weaken as the daughters aged. For presence of prenatal or postpartum depression only, all associations weakened with adjustment, with the exception of prenatal depression only and nocturia among daughters at 9 years (aOR 1.30, 95% CI 1.1-1.6), and postpartum depression only with daughters’ daytime wetting at 6 and 7 years (aOR 2.11, 95% CI 1.4-3.1; aOR 1.83, 95% CI 1.1-2.9, respectively).

## Discussion

This study contributes to an important, yet understudied knowledge gap in urologic research. In mother-daughter dyads participating in ALSPAC, we detected an association between screening positive for prenatal and postpartum depression and LUTS in primary school-age daughters. The potential LUTS associations for these daughters may be an important opportunity for LUTS prevention during childhood. Pediatricians, primary care clinicians, and WOC nurses are encouraged to raise their awareness of the increased possibility of LUTS in daughters of women with a maternal depression history, especially early in childhood. In addition, WOC and other nurses who participate in continence care of children have an opportunity to inquire about maternal mental health which may also help detect ongoing maternal depression remote from pregnancy. This study expands on previous findings by showing that positive maternal depression screening is associated with urinary urgency and nocturia, and not just urinary incontinence, in the daughters; these associations weakened as the daughters grew older.

The biologic mechanism for the association between maternal depression and daughter’s risk of LUTS is unknown. Prenatal programming of the fetal hypothalamic pituitary adrenal axis (HPAA) may be one mechanism by which maternal mood during pregnancy exerts an enduring effect on the neurodevelopment of the offspring.^26^ Shared genetic susceptibility for negative affect in the mother-daughter dyads is an alternative explanation for the observed associations, since there is evidence for a link between affective symptoms and the development of LUTS.^1,27^ Further research is needed to determine whether there is a causal association between affective symptoms and LUTS. However, it is unlikely that a biological mechanism occurs exclusively during pregnancy. Our finding that a positive postpartum maternal depression screen was also associated with LUTS in their daughters challenges the assumption of a pregnancy specific mechanism and raises consideration of the contextual or potentially environmental contribution to the associations.

The timing of exposure to postpartum depression (21 months) was chosen because it coincides with the period when many ALSPAC mothers reported that they had initiated toilet training.^17^ It is possible that exposure to maternal depression during this important developmental transition affects the attainment of continence through disrupted or inadequate toilet training strategies. Mothers with depression may benefit from interventions designed to help them cope with the demands of toilet training. While the association between maternal depression and LUTS in daughters was even stronger in participants who screened positive for both prenatal and postpartum depression, a dose-effect model was not confirmed across all time points. However, the hypothesis of a dose-effect model remains plausible and should be tested in the future.

### Strengths and Limitations

This study has multiple strengths, including its prospective and repeated collection of screening measures for maternal depression, its decade-long follow-up and repeated assessment of multiple LUTS in their offspring, and its large sample size. Together, these unique strengths allowed us to examine the individual and cumulative effects of maternal prenatal and postpartum depression on school-age daughters prospectively.

This study has several limitations. First, as with all observational studies, these findings cannot confirm a causal association. Second, the study recruited women living in a defined area in the South West of England, the majority of whom were White; thus the findings may not be generalizable to other populations. Third, like many large, population-based surveys, the ALSPAC cohort has experienced attrition; families lost to follow-up were more likely to come from socio-economically disadvantaged backgrounds than those with complete data.^16^ Fourth, data collection for the ALSPAC study initiated in 1991 and 1992, since which time there have been advances in treatment for maternal depression. Nonetheless, our findings are relevant because they extend the rationale for detection and treatment during pregnancy through several years following birth because of the potential effects on the daughter. Specifically, more than a third (36.3%) of women categorized as having prenatal depression were still categorized as depressed nearly two years after birth. Fifth, depression was assessed by self-report rather than clinical diagnoses; the selection of an EPDS cutoff score of 12/13 results in high diagnostic specificity (0.95), but lower sensitivity (0.66).^20^ As ongoing treatment for depression was not assessed, we are unable to comment on the effectiveness of detection or treatment during pregnancy. Sixth, we did not assess child maturational level or emotional/behavioral problems. These child variables may interact with maternal depression in the postpartum period to influence the risk of LUTS in the daughter, or be on the causal pathway from maternal depression (prenatal or postpartum) to LUTS. The intersectionality of maternal depression during the childbearing years and subsequent parenting, stress, and socialization of the infant/child/daughter also should be considered in future research. Given the possibility that maternal depression may influence how mothers assess their children’s LUTS or urinary function, observer bias could influence the findings as mothers were the principal reporter of pediatric LUTS.

## Conclusion

The duration of maternal depression influenced childhood urinary health, with cumulative exposure increasing the association of multiple LUTS in their daughters. Existing efforts to identify and treat prenatal and postpartum depression are further justified by the potential LUTS risks for daughters. Prevention strategies should reflect an understanding of potential biologic and environmental mechanisms through which maternal depression may influence childhood LUTS. Future studies should consider contextual assessment, infant attachment trajectories, more diverse samples, and prevention strategies to influence these areas as potential intervention opportunities.

## Supplementary Material

Supplementary Material

## Figures and Tables

**Figure 1:**
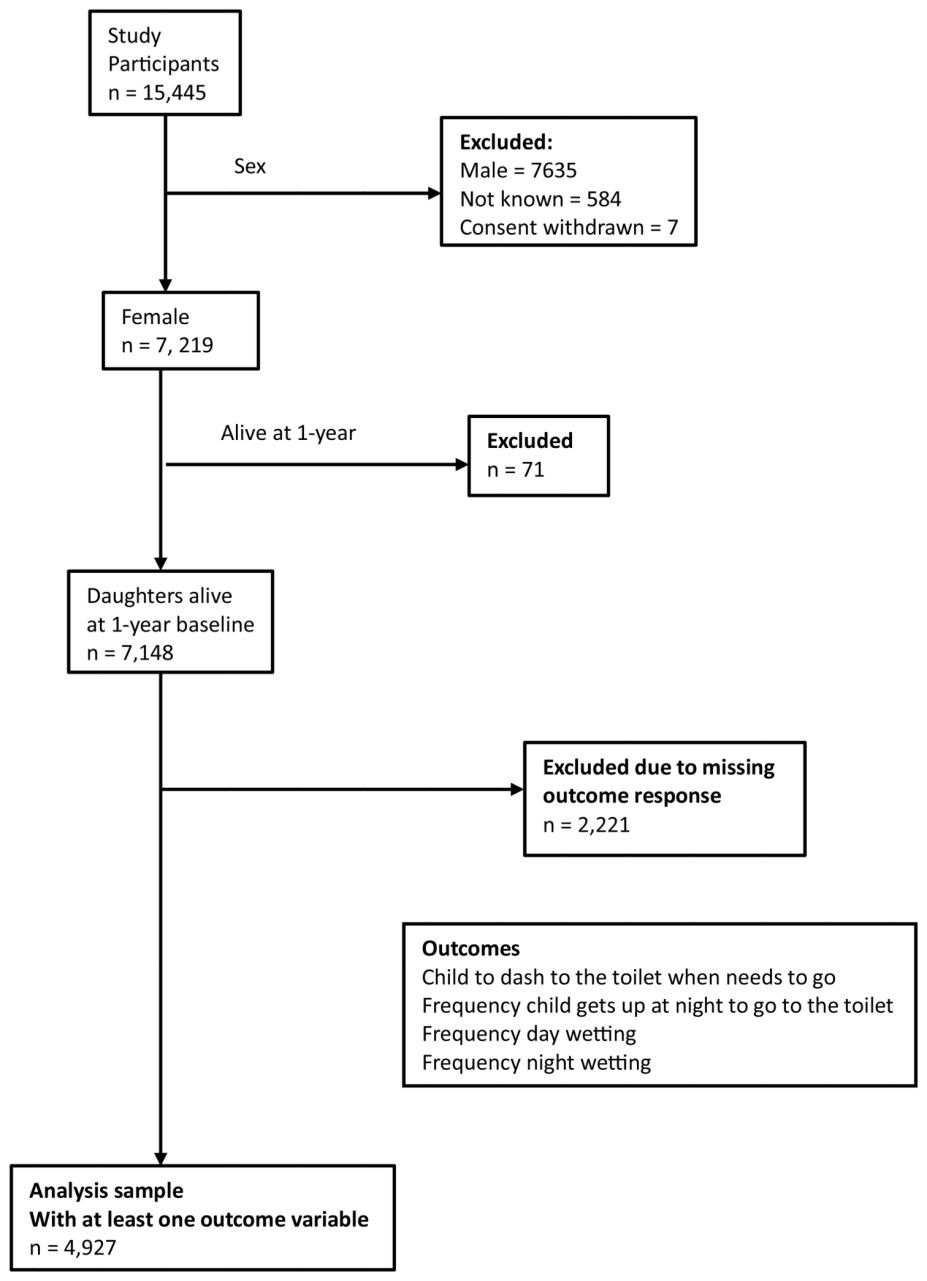
Study Flowchart

**Table 1:** Sample Characteristics

ALSPAC Characteristics			
	Pre-Imputation Sample (at least one outcome)		Imputed Sample (averaged from 20 sets)
	n (%)	n Total n=4927	n (%) 4927
***Maternal depression***			
None	3014 (76.8)	3922	3739 (75.9)
Prenatal Only	546 (13.9)		645 (13.1)
Postpartum Only	140 (3.6)		177 (3.6)
Both prenatal and postpartum	222 (5.7)		367 (7.4)
***Lower Urinary Tract Symptoms (LUTS)***			
**Age 6**			
Urinary urgency (has to go straight away vs. can wait for any duration of time)	399 (9.8)	4064	514 (10.4)
Nocturia (gets up 1 or more times at night vs. not at all)	1018 (25.5)	3986	1308 (26.5)
Daytime wetting			
Never	3620 (88.4)	4097	4344 (88.2)
Occasional (< 1 per week) or about once a week	436 (10.6)		513 (10.4)
2 times a week or more	41 (1.0)		71 (1.4)
Nighttime wetting			
Never	3516 (85.7)	4102	4222 (85.7)
Weekly (1-5 times a week)	527 (12.8)		629 (12.8)
Daily (nearly every day or oncer per day)	59 (1.4)		76 (1.5)
**Age 7**			
Urinary urgency (has to go straight away vs. can wait for any duration of time)	328 (8.3)	3973	450 (9.1)
Nocturia (gets up 1 or more times at night vs. not at all)	909 (23.4)	3879	1241 (25.2)
Daytime wetting			
Never	3634 (91.1)	3987	4459 (90.5)
Occasional (< 1 per week) or about once a week	302 (7.6)		389 (7.9)
2 times a week or more	51 (1.3)		80 (1.6)
Nighttime wetting			
Never	3533 (89.5)	3948	4355 (88.4)
Weekly (1-5 times a week)	383 (9.7)		522 (10.6)
Daily (nearly every day or oncer per day)	32 (0.8)		50 (1.0)
**Age 9**			
Urinary urgency (has to go straight away vs. can wait for any duration of time)	282 (7.1)	3975	411 (8.3)
Nocturia (gets up 1 or more times at night vs. not at all)	937 (24.5)	3823	1291 (26.2)
Daytime wetting			
Never	3760 (94.2)	3992	4590 (93.2)
Occasional (< 1 per week) or about once a week	211 (5.3)		305 (6.2)
2 times a week or more	21 (0.5)		32 (0.7)
Nighttime wetting			
Never	3755 (94.0)	3993	4563 (92.6)
Weekly (1-5 times a week)	227 (5.7)		337 (6.8)
Daily (nearly every day or once per day)	11 (0.3)		27 (0.5)
***Covariates***			
Manual social class (vs nonmanual)	719 (16.6)	4330	831 (16.9)
Home ownership	3636 (79.3)	4583	3832 (77.8)
Family size >=3	191 (4.2)	4539	301 (6.1)
Financial difficulties (a lot or fairly vs mild to none)	296 (7.0)	4236	395 (8.0)
Maternal education			
Low	1139 (25.0)	4557	1318 (26.8)
Medium	1596 (35.0)		1678 (34.1)
High	1822 (40.0)		1930 (39.2)

**Table 2:** Association between maternal depression and lower urinary tract symptoms in their school age daughters^1^

Outcome^2^	Odds Ratio (95% Confidence Interval)					
	Unadjusted			Adjusted^1^		
	Age 6	Age 7	Age 9	Age 6	Age 7	Age 9
Urinary urgency						
Prenatal depression only	1.50 (1.1 - 2.0)*	1.43 (1.1 - 1.9)*	1.38 (1.0 - 1.9)	1.32(1.0-1.8)	1.26 (0.9-1.7)	1.22 (0.9-1.7)
Postpartum depression only	1.49 (0.9 - 2.5)	1.89 (1.1 - 3.2)*	1.46 (0.8 - 2.6)	1.42 (0.9-2.5)	1.76 (1.1-3.0)	1.40 (0.8-2.5)
Both prenatal and postpartum depression	3.18 (2.3 - 4.4)**	3.50 (2.5 - 5.0)**	2.69 (1.9 - 3.7)**	2.33 (1.7-3.3)**	2.50 (1.7-3.7)**	1.99(1.4-2.9)*
Nocturia						
Prenatal depression only	1.35 (1.1 - 1.6)*	1.51 (1.2 - 1.8)**	1.50 (1.2 - 1.8)**	1.19 (1.0-1.5)	1.27 (1.0-1.6)	1.30 (1.1-1.6)*
Postpartum depression only	1.39 (1.0 - 2.0)	1.36 (0.9 - 1.9)	1.33 (0.9 - 2.0)	1.36 (1.0-1.9)	1.28 (0.9-1.8)	1.28 (0.9-1.9)
Both prenatal and postpartum depression	2.40 (1.8 - 3.1)**	2.60 (2.0 - 3.3)**	2.19 (1.7 - 2.8)**	1.97 (1.5-2.6)**	1.87 (1.4-2.5)**	1.67 (1.3-2.2)**
Daytime wetting						
Prenatal depression only	1.14 (0.9 - 1.5)	1.10 (0.8 - 1.5)	1.50 (1.1 - 2.1)*	1.03 (0.8-1.4)	0.95 (0.7-1.3)	1.28 (0.9-1.8)
Postpartum depression only	2.26 (1.5 - 3.4)**	2.00 (1.3 - 3.2)*	1.37 (0.7 - 2.8)	2.11 (1.4-3.1)**	1.83 (1.1-2.9)*	1.26 (0.6-2.5)
Both prenatal and postpartum depression	2.54 (1.8 - 3.6)**	2.57 (1.7 - 3.8)**	2.13 (1.4 - 3.2)**	1.99 (1.4-2.8)**	1.81 (1.2-2.8)*	1.51 (1.0-2.4)
Nightime wetting						
Prenatal depression only	1.23 (1.0 - 1.6)	1.31 (1.0 - 1.7)	1.43 (1.0 - 2.0)	1.11 (0.9-1.4)	1.15 (0.9-1.5)	1.21 (0.9-1.7)
Postpartum depression only	1.33 (0.9 - 2.0)	1.31 (0.8 - 2.1)	1.99 (1.2 - 3.3)*	1.23 (0.8-1.9)	1.19 (0.7-2.0)	1.83 (1.1-3.1)
Both prenatal and postpartum depression	2.53 (1.9 - 3.4)**	2.33 (1.7 - 3.2)**	2.45 (1.7 - 3.5)**	1.95 (1.4-2.7)**	1.63 (1.1-2.3)*	1.69 (1.1.-2.5)

1 Controlling for social class, maternal educational attainment, home ownership status, financial difficulties, and family size.

2 Reference category: no depression. Women were classified as having experienced prenatal depression if they scored ≥13 on the EPDS at 18 and/or 32 weeks gestation. Postpartum depression data were collected 21 months after birth.

* False Discovery Rate p<0.05;

** p<0.001
