# Supplementary material for: Association between maternal depression and lower urinary tract symptoms in their primary school-age daughters: A birth cohort study
Source: J Wound Ostomy Continence Nurs. Author manuscript; Available in PMC 2024 Feb 15. (PMC10794027; doi:10.1097/WON.0000000000001039)
Supplement: Supplementary Material [file EMS188634-supplement-Supplementary_Material.docx]

**Supplemental Digital Content**

**APPENDIX**

**Association between maternal depression and lower urinary tract symptoms in their school age daughters(Complete case analysis, i.e., without imputation. n=2442)**

|  | | | | | | |
| --- | --- | --- | --- | --- | --- | --- |
|  | **Odds Ratio (95% Confidence Interval)** | | | | | |
| **Outcome^2^** | **Unadjusted** | | | **Adjusted^1^** | | |
|  | **Age 6** | **Age 7** | **Age 9** | **Age 6** | **Age 7** | **Age 9** |
| **Urinary urgency** |  |  |  |  |  |  |
| Prenatal depression Only | 1.50 (1.0 - 2.3) | 1.24 (0.8 - 2.0) | 1.07 (0.7 - 1.8) | 1.40 (0.9 - 2.2) | 1.29 (0.8 - 2.1) | 1.10 (0.7 - 1.9) |
| Postpartum depression Only | 1.30 (0.6 - 2.6) | 1.57 (0.8 - 3.2) | 1.40 (0.7 - 3.0) | 1.24 (0.6 - 2.6) | 1.46 (0.7 - 3.1) | 1.31 (0.6 - 2.9) |
| Both prenatal and postpartum depression | 2.03 (1.1 - 3.6)* | 1.71 (0.9 - 3.3) | 1.40 (0.7 - 2.8) | 2.04 (1.1 - 3.7)* | 1.42 (0.7 - 3.0) | 1.44 (0.7 - 3.0) |
| **Nocturia** |  |  |  |  |  |  |
| Prenatal depression Only | 1.42 (1.1 - 1.9)* | 1.56 (1.2 - 2.1)* | 1.40 (1.1 - 1.9)* | 1.31 (1.0 - 1.8) | 1.29 (0.9 - 1.8) | 1.29 (0.9 - 1.8) |
| Postpartum depression Only | 1.09 (0.7 - 1.8) | 0.98 (0.6 - 1.7) | 1.13 (0.7 - 1.9) | 1.14 (0.7 - 1.9) | 0.96 (0.6 - 1.7) | 1.18 (0.7 - 2.0) |
| Both prenatal and postpartum depression | 2.00 (1.3 - 3.0)** | 2.52 (1.7 - 3.8)** | 1.77 (1.2 - 2.7)* | 2.02 (1.3 - 3.1)* | 2.27 (1.5 - 3.5)** | 1.71 (1.1 - 2.7)* |
| **Daytime wetting** |  |  |  |  |  |  |
| Prenatal depression Only | 0.91 (0.6 - 1.4) | 1.00 (0.6 - 1.6) | 1.28 (0.8 - 2.1) | 0.94 (0.6 - 1.4) | 1.05 (0.6 - 1.7) | 1.13 (0.6 - 2.0) |
| Postpartum depression Only | 1.63 (0.9 - 2.8) | 2.24 (1.3 - 4.0)* | 1.06 (0.4 - 2.7) | 1.63 (0.9 - 2.9) | 2.04 (1.1 - 3.7)* | 1.02 (0.4 - 2.6) |
| Both prenatal and postpartum depression | 1.33 (0.8 - 2.3) | 0.98 (0.5 - 2.0) | 1.32 (0.6 - 2.9) | 1.15 (0.6 - 2.1) | 0.77 (0.3 - 1.7) | 0.92 (0.4 - 2.2) |
| **Nighttime wetting** |  |  |  |  |  |  |
| Prenatal depression Only | 1.10 (0.8 - 1.6) | 1.35 (0.9 - 2.0) | 1.22 (0.7 - 2.1) | 1.11 (0.7 - 1.6) | 1.35 (0.9 - 2.0) | 1.16 (0.6 - 2.1) |
| Postpartum depression Only | 0.81 (0.4 - 1.6) | 0.93 (0.4 - 2.0) | 2.16 (1.1 - 4.4)* | 0.85 (0.4 - 1.7) | 0.95 (0.4 - 2.0) | 2.33 (1.1 - 4.8)* |
| Both prenatal and postpartum depression | 2.30 (1.5 - 3.6)** | 1.63 (0.9 - 2.9) | 1.89 (0.9 - 3.9) | 2.30 (1.4 - 3.7)** | 1.63 (0.9 - 2.9) | 1.87 (0.9 - 3.9) |

^1^Controlling for social class, maternal educational attainment, home ownership status, financial difficulties, and family size.

^2^Reference category: no depression. Women were classified as having experienced prenatal depression if they scored ≥13 on the EPDS at 18 and/or 32 weeks gestation. Postpartum depression data were collected 21 months after birth.

* p<0.05, **p<0.001
